# The impact of the confinement of reactants on the metal distribution in bimetallic nanoparticles synthesized in reverse micelles

**DOI:** 10.3762/bjnano.5.206

**Published:** 2014-11-04

**Authors:** Concha Tojo, Elena González, Nuria Vila-Romeu

**Affiliations:** 1 Physical Chemistry Department, Faculty of Chemistry, University of Vigo, 36310 Vigo, Spain; 2 Physical Chemistry Department, Faculty of Sciences, University of Vigo, 32004 Ourense, Spain

**Keywords:** bimetallic nanoparticles, intermicellar exchange rate, microemulsion simulation

## Abstract

A kinetic study on the formation of bimetallic nanoparticles in microemulsions was carried out by computer simulation. A comprehensive analysis of the resulting nanostructures was performed regarding the influence of intermicellar exchange on reactivity*.* The objects of this study were metals having a difference in standard reduction potential of about 0.2–0.3 V. Relatively flexible microemulsions were employed and the concentration of the reactants was kept constant, while the reaction rate of each metal was monitored as a function of time using different reactant proportions. It was demonstrated that the reaction rates depend not only on the chemical reduction rate, but also on the intermicellar exchange rate. Furthermore, intermicellar exchange causes the accumulation of slower precursors inside the micelles, which favors chemical reduction. As a consequence, slower reduction rates strongly correlate with the number of reactants in this confined media. On the contrary, faster reduction rates are limited by the intermicellar exchange rate and not the number of reactants inside the micelles. As a result, different precursor proportions lead to different sequences of metal reduction, and thus the arrangement of the two metals in the nanostructure can be manipulated.

## Introduction

The advancement in the field of nanotechnology relies on the improvement in nanoparticle preparation techniques. Thus, research has been targeted at the development of different synthetic technologies to achieve control over the composition, structure and shape of nanoparticles. Nowadays there are many methods available to synthesize nanoparticles. From the pioneering research of Boutonett et al. [1], synthesis using microemulsions became a frequently used technique [2–10]. This method offers many advantages with respect to other techniques, specifically, the possibility to prepare different types of materials using simple equipment while obtaining very small particles with a narrow size distribution whose composition is well-controlled. To synthesize nanoparticles by this method, one reactant is solved inside the droplets of a microemulsion, and another reactant is solved inside the droplets of a second microemulsion. After mixing, the microemulsions reactants can cross intermicellar channels and come into contact when they are located inside the same micelle due to micelle collisions and coalescence. The chemical reaction can then take place to form precipitates of nanometric size, which remain confined to the interior of reverse micelles. This approach has been used to prepare a variety of nanomaterials [6,11–15] that often display better catalytic activity than nano-catalysts prepared by other methods [10].

More complex structures, such as bimetallic nanoparticles, have also been prepared via microemulsions [11,14,16–19]. The combination of two different metallic atoms can result in the electronic coupling between the individual metals in the resulting particle, causing bimetallic nanoparticles to show different behavior from monometallic ones. The synthesis of bimetallic nanoparticles has drawn increasing attention in the field of nanotechnology, because their physical and chemical properties are composition-dependent [15,20–23]. Specifically, in the field of catalysis, bimetallic nanoparticles exhibit significantly increased catalytic behavior in comparison to monometallic nanoparticles [24]. Of interest is the controlled segregation and the extent of alloying of the two metals, since these factors profoundly affect catalytic activity and selectivity. Most of the catalytic reactions are structure sensitive, and bimetallic nanoparticles offer the opportunity to tune the catalytic properties by modifying the composition distribution [25]. Although it is of enormous importance to control the metal distribution in nanoparticles, predicting this is very difficult [26]. In addition, even in the case of bimetallic nanoparticles having similar compositions, different atomic distributions have been found depending on the preparation method. The accurate control of the bimetallic nanostructure remains a challenge. The design of catalysts for improved activity and selectivity must be based on a sufficiently good understanding of the reaction mechanism.

For a better insight into the metal distribution in a bimetallic nanoparticle synthesized in microemulsions, three aspects must be taken into consideration. First, it is believed that the nucleus develops into a particle by building up new layers, so the sequence of deposition of the metals determines the metal distribution. Second, this sequence is strongly dependent on the reduction rates of the two metal ions. Thus, if one metal reduces faster than the other, it will form the core. The metal which is reduced more slowly will be deposited onto this core, forming the outer layers, which results in a core–shell nanostructure. In contrast, a small difference between reduction rates of the two metals leads to an alloy. Third, this result was obtained in homogeneous reaction media. It was extended to synthesis inside micelles without taking into account the heterogeneity of reaction media. It is understood that reverse micelles are reaction vessels in which classical assumptions cannot always be used. For example, it was demonstrated that the concept as simple as the classical definition of pH cannot be applied in the interior of reverse micelles [27–28]. Previous simulation studies concluded that the structure of nanoparticles obtained in reverse micelles is determined by the difference in the reduction rates only if both reductions occur at the same rate or if reductions have very different rates [23,29]. That is, in these two extreme cases, compartmentalization of the reaction media cannot modify the metal arrangement. However, a vast majority of bimetallic systems belong to the large category between these extremes in which the metal segregation depends on the microemulsion dynamics.

With the exception of the existing studies relating nanoparticle properties to microemulsion composition [2–3,30–33], there is a gap regarding the impact of microemulsion dynamics on metal segregation in bimetallic nanoparticles. The synthesis of simple [34–35] and bimetallic [29,36] nanoparticles in microemulsions has been previously studied. The research at hand is focused on the rates of the chemical reaction inside micelles, which may provide fundamental knowledge on how the metal distribution in the nanostructure can be modified. The hypothesis is that the resulting nanostructure is due to the particular combination of three main factors: the intermicellar exchange rate (determined by microemulsion composition), the difference in reduction rate of both metals, and the amount of metal precursors inside the micelles. Together, this leads to a particular sequence of deposition of the metals, which in turn determines the metal distribution in the final nanoparticle. This article reports a simulation study, which analyzes how the intermicellar exchange influences reactivity, and provides a comprehensive analysis of the resulting nanostructures.

To verify the validity of the model predictions, simulation results of the arrangement of Au/Pt nanoparticles prepared in microemulsions were compared with Au/Pt nanoparticles synthesized using the same conditions of the simulation studies. The successful agreement between the theoretical and experimental STEM (scanning transmission electron microscopy) profiles confirms the validity of the simulation model [37]. The Au/Pt bimetallic system was successfully simulated by employing a reduction rate ratio of v_Au_/v_Pt_ ≈ 10. This value can be associated with bimetallic couples with a difference in standard reduction potential of about Δε ≈ 0.2–0.3 V [29]. These results can be generalized to other bimetallic nanoparticles whose Δε is within this range.

To design a synthesis route to obtain a bimetallic particle with a particular nanostructure, one must manipulate the metal distribution by varying the experimental conditions. It would be highly beneficial if changes in the metal arrangement could be obtained by simply changing the proportion of reactants. With the final goal of tuning the conditions for synthesizing specific bimetallic structures, we studied whether the metal distribution could be modified by a change in the proportion of reactants.

## Simulation Procedure

The main strategy behind the one pot method for the synthesis of Au/Pt bimetallic nanoparticles using microemulsions involves mixing three microemulsions, each containing one reactant (the two metal salts and reducing agent). After mixing, the micelles move and collide, allowing reactants to come into contact with one another due to material transfer between colliding droplets. It is assumed that this intermicellar exchange occurs when a collision between two micelles results in the merger of the micelles, establishing a water channel between them. When one of the two metal salts ([AuCl_4_]^−^or [PtCl_6_]^2−^ for the preparation of Au/Pt particles) and the reducing agent (e.g., hydrazine) are located in the same micelle, the chemical reduction takes place inside the reverse micelle to obtain metal atoms (Au or Pt). That is, the droplets of the microemulsion can be conceived as tiny compartments or nano-reactors. The isolation of each nanoparticle inside a micelle avoids nanoparticle aggregation with particles located in nearby micelles. In this way, microemulsion droplets act as ideal templates, providing a compartmentalized reaction environment. The simulation model employed in this research attempts to simulate the kinetic course of the chemical reaction in these nanoreactors. In order to study the metal distribution in the final nanoparticle, the order of the metal reduction inside each micelle is stored and analyzed at the end of the synthesis.

### Reaction media description

The microemulsion structure is assumed as spherical micelles in a continuous oil phase. To reproduce this heterogeneous media, the microemulsion is defined as a set of micelles randomly located in a three dimensional lattice. Each simulation run starts with three different sets of micelles randomly distributed: micelles carrying Au salt (M-Au), Pt salt (M-Pt) and reducing agent (M-R). The volume fraction occupied by micelles is set at φ = 10%. Each micelle can act as a nanoreactor during nanoparticle synthesis. Thus, although initially each microemulsion carries only one kind of reactant, as the synthesis takes place each micelle may have species coexisting together: reactants (faster reduction metal salt [AuCl_4_]^−^, slower reduction metal salt [PtCl_6_]^2−^ and reducing agent R), free metal products (Au and Pt), and growing particles (aggregates composed by Au and Pt atoms).

The movement of micelles is assumed to be governed by Brownian motion. Two different strategies have been tested to simulate the movement and collisions of micelles. In the first one, micelles diffuse on a square lattice by performing random walks to contiguous lattice sites [38]. The movement obeys the exclusion principle (a lattice site can be occupied by only one micelle at the same time) and cyclic boundary conditions were implemented. A collision between micelles takes place when two micelles occupy two contiguous lattice sites. In the second method, two micelles are randomly selected to collide. Because of collision, micelles fuse to form a short-lived dimer and a water channel is established between them [39], allowing the exchange of material content. After collision, the micelles redisperse. To save computation time all collision are assumed to be effective. Similar results were obtained by using both collision models. The latter requires less computation time, thus it was implemented in this study.

### Microemulsion composition

Microemulsions consist of water nano-droplets dispersed in a continuous oil medium. The dispersion is stabilized by a monolayer of surfactant which accumulates at the oil–water interface. The flexibility of the surfactant film surrounding micelles is a parameter associated with the interfacial curvature, which depends on the interactions on both sides of the interface. The flexibility is dictated by the microemulsion composition (mainly by the surfactant), and is directly related to the facility with which intermicellar channels can be established. The intermicellar exchange of material takes place through the intermicellar channel, thus the kinetics of the nanoparticle formation will strongly depend on the channel feature.

Two aspects must be taken into account in order to establish how surfactant film flexibility is included in the simulation model. First, the material exchange between micelles will only be possible if the dimer remains intact for a sufficient amount of time. The longer that two colliding micelles stay together, the greater the number of species that can be exchanged during a collision. Therefore, dimer stability is directly related to the intermicellar exchange rate. Second, the intermicelar channel diameter restricts the size of the particles capable of crossing the channel. It is also related to the microemulsion composition, because the more flexible the film, the larger the channel size. That is, a more flexible film allows the exchange of larger particles than a rigid film. The most important factor determining the intermicellar exchange of isolated species, such as reactants and non-aggregated metallic atoms, is the dimer stability [38]. This is because free species traverse the channel one by one. Thus the channel size would not be important in this case. On the contrary, the channel size is the most important determining factor if the exchanged species is a growing particle (an aggregate of metal atoms). From this perspecive, the flexibility of the surfactant film is introduced in the model by means of two simulation parameters: the exchange parameter, *k_ex_*, which dictates the exchange protocol of free species (see below), and the flexibility parameter, *f*, which restricts the size of exchanged particles. Both parameters must increase together, because a flexible film implies that a larger particle can be transferred (high *f*) and that intermicellar exchange is faster (high *k_ex_*). Experimental results obtained in a rigid microemulsion, such as AOT (sodium bis(2-ethylhexyl) sulfosuccinate)/*n*-heptane/water, were succesful compared to simulation data where the flexibility was characterized by *f* = 5 and *k_ex_* = 1 [34]. Nanoparticles obtained using a more flexible microemulsion, such as isooctane/tergitol/water, were successfully reproduced by simulation data using *f* = 30 and *k_ex_* = 5 [37].

### Initial concentration inside micelles

Initially, each reactant ([AuCl_4_]^−^, [PtCl_6_]^2−^ and R) is distributed throughout the micelles of the corresponding microemulsion according to the Poisson distribution as:

[1]
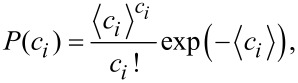


where *i* =[AuCl_4_]^−^, [PtCl_6_]^2−^ or R which is the metal salt or reducing agent, *c_i_* is the number of reactants per micelle, and *P*(*c_i_*) is the probability that a micelle carries *c_i_* reactants whose average occupancy is 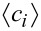. We present results using an average value of 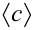 = 64 reactants in a micelle, which corresponds to 0.40 M. Molar concentration was calculated considering the droplet radius, *r*, of a 75% isooctane/20% tergitol/5% water microemulsion (*r* = 4 nm as obtained by DLS (dynamic light scattering)). From this radius, and assuming a spherical shape (*V*_micelle_ = 4/3π *r^3^*), the molar concentration of a micelle containing 64 atoms is calculated as:

[2]
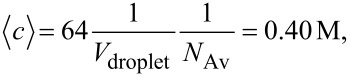


where *N*_Av_ is Avogadro’s number. In order to study the influence of the proportion of reactants, the average concentration is kept constant (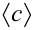 = 64 reactants per micelle) and the proportion of each reactant was varied as follows: 12.5% Au, 
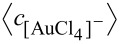
 = 16, 
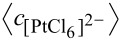
 = 112; 25% Au, 
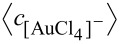
 = 32, 
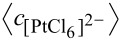
 = 96; 37.5% Au, 
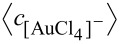
 = 48, 
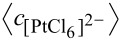
 = 80; 50% Au, 
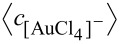
 = 64, 
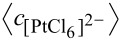
 = 64; 62.5% Au, 
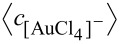
 = 80, 
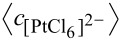
 = 48; 75% Au, 
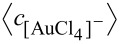
 = 96, 
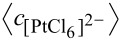
 = 32; 87.5% Au, 
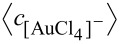
 = 112, 
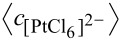
 = 16. The reducing agent concentration 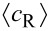 was always double that of the average concentration of the metal precursors (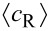 = 128 molecules of hydrazine per micelle). For convenience, Table 1 provides a summary of the acronyms and abbreviations used throughout this article.

**Table 1:** Glossary of acronyms and abbreviations used in this article.

[AuCl_4_]^−^	faster metal precursor (Au salt)
[PtCl_6_]^2−^	slower metal precursor (Pt salt)
R	reducing agent
M-Au	micelle containing Au salt
M-Pt	micelle containing Pt salt
M-R	micelle containing reducing agent
Au	faster metal atom (reduction product of metal precursor [AuCl_4_]^−^)
Pt	slower metal atom (reduction product of metal precursor [PtCl_6_]^2−^)
*P*(*c_i_*)	probability that a micelle carries *c_i_* reactants ([AuCl_4_]^−^, [PtCl_6_]^2−^ or R)
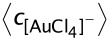	mean number of metal precursor [AuCl_4_]^−^ at the beginning
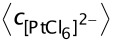	mean number of metal precursor [PtCl_6_]^2−^ at the beginning
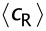	mean number of reduced molecules R at the beginning
*k_ex_*	exchange parameter: number of free units ([AuCl_4_]^−^, [PtCl_6_]^2−^, Au, Pt, R) which could be transferred during a collision
v_Au_	faster chemical reduction rate: percentage of [AuCl_4_]^−^ inside the colliding droplets which gives rise to products
v_Pt_	slower chemical reduction rate: percentage of [PtCl_6_]^2−^ inside the colliding droplets which gives rise to products
n_Au_	number of Au atoms (reduction product of faster metal precursor [AuCl_4_]^−^)
n_Pt_	number of Pt atoms (reduction product of slower metal precursor [PtCl_6_]^2−^)
dn_Au_/dt	Au reaction rate; calculated from simulation data
dn_Pt_/dt	Pt reaction rate; calculated from simulation data
*n*_A_*	critical nucleus size: minimum number of A atoms required to form a stable nucleus
*n*_B_*	critical nucleus size: minimum number of B atoms required to form a stable nucleus
*n*_A-B_*	heterogeneous critical size: minimum number of A and B required to form a stable nucleus
*f*	film flexibility: maximum particle size for transfer between droplets
*q*	maximum number of metal atoms which can be carried by a droplet

### Time unit base

The time unit is one Monte Carlo step, which is defined as follows. One Monte Carlo step starts when 10% of the micelles are chosen to collide at random. The micelles then fuse and material exchange may take place. The nature and quantity of species inside the chosen micelles can be modified according to the exchange criteria described below. Once the composition inside both micelles is updated, one Monte Carlo step (mcs) is completed. The composition inside each micelle is stored step by step, because the sequence of the metal reduction is decisive in predicting the final nanoparticle structure. These results monitor the evolution of particle distribution as a function of time. One simulation run is finished when the composition of every particle inside all micelles remains constant.

#### Intermicellar exchange protocol of reactants and metal atoms

Species can be transferred between micelles during the short-lived dimer formation. This transfer is closely related to the intermicellar exchange rate, because the faster the rate, the more species that can traverse the dimer channel during a collision. The exchange criterium will be the concentration gradient, that is, species flow from a region (micelle) of higher concentration to one of lower concentration until the concentration becomes equal. A simulation parameter *k_ex_* is included to restrict the maximum number of reactants ([AuCl_4_]^−^, [PtCl_6_]^2−^ or R) which can be exchanged between micelles during a collision. If the higher occupied micelle contains a number of molecules greater than *k_ex_*, then the maximum number of reactant molecules that can cross the channel towards the micelles containing less reactants is *k_ex_*. If the number of reactants to be exchanged is lower than *k_ex_*, reactants are redistributed until the concentration inside both colliding micelles becomes equal after collision.

It is assumed that *k_ex_* is mainly determined by the microemulsion composition [39–42] and the nature of the material is less important. Thus, although the characteristics of the species traversing the channel could modify the intermicellar exchange rate, a single value of *k_ex_* was used in this investigation (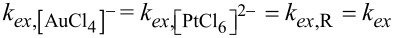).

#### Chemical reduction rates

Due to the redistribution of material between the micelles, one metal salt and the reducer can be located inside the same micelle in order for chemical reduction to take place. The reduction potentials of the two metal salts, [AuCl_4_]^−^ and [PtCl_6_]^2−^, are different, so both chemical reductions will occur at different rates. The two metal salts are reduced according to the simulation parameter, v*_i_*, where *i* corresponds to the metal salt, [AuCl_4_]^−^ or [PtCl_6_]^2−^. This parameter represents the chemical reduction rate as the probability of obtaining one reduced metal atom from each metal salt/reducing agent molecule pair available in the micelle. Because of the faster reduction rate of Au, it is assigned a value of v_Au_ = 1, that is, 100% of reactants inside micelles gives rise to products. This implies that the reaction is completed and only Au atoms and excess reactants (either [AuCl_4_]^−^ or R) are to be distributed to daughter micelles. Hence, the reactants [AuCl_4_]^−^ and R will not coexist in a micelle. In order to consider different reactions rates, the probability of reactants located in the micelle that reduces to metal atoms can be decreased. In this study we present results where this parameter is fixed at v_Au_/v_Pt_ = 10. This means that only one of every ten pairs of [PtCl_6_]^2−^ and a reducing agent available in the micelle produces Pt atoms. The remainder of the [PtCl_6_]^2−^ and reducing agent that did not react remains in the micelle and can be exchanged or can react in a future collision. When both reduction reactions are possible because all three reactants ([AuCl_4_]^−^, [PtCl_6_]^2−^ and R) are located inside the same micelle at the same time, both reductions are allowed to take place during the same collision.

#### Nucleation

Nucleation is the process by which atoms (ions or molecules), which were initially isolated in solution, arrange to form a thermodynamically stable nucleus. The nucleus grows as more atoms are deposited onto it. A new phase (nucleus) begins to form from random fluctuations. Because a nucleation event is hindered by energy barriers, only fluctuations which overcome these nucleation barriers can give rise to the formation of a nucleus capable of further growth [43]. Classical nucleation theory establishes the existence of a critical nucleus size from which a nucleus will grow instead of dissolving. A nucleus smaller than this critical size will spontaneously dissolve. In microemulsions, nucleation similarly requires the presence of enough atoms to exceed the critical nucleation size inside the same micelle [44]. Nucleation is included in the model by means of the variable critical nucleus, n*, which is compared with the actual amount of metal atoms inside the same micelle. If it is smaller than n*, the atoms remain free (non-aggregated) inside the micelle because the nucleus is considered to be unstable and will disintegrate to produce isolated atoms. This implies that atoms can be exchanged during a subsequent collision subject to *k_ex_* parameter. However, if the number of reduced atoms located inside the same micelle exceeds n*, all atoms gather producing a stable nucleus capable of further growth. The exchange of nucleii between colliding micelles depends on the intermicellar channel size, since an aggregate of atoms must be exchanged as a whole. This type of material exchange is governed by the film flexibility parameter, *f* (see below).

The total Gibbs energy of an atomic arrangement determines the alloying ability of a bimetallic nanoparticle A-B. It depends on composition because of the different atomic binding energy of A-A, B-B, and A-B species. These different binding energies (A-A, B-B, and A-B) dictate the minimum size that must be reached by atoms for nucleation depending on the composition. This phenomena is included in the simulation model by means of the inclusion of three critical nucleus numbers (*n*_A_*, *n*_B_* and *n*_A-B_*). Once the critical number is exceeded, the cluster grows by deposition of all metal atoms (either Au or Pt) located inside the same micelle. It is assumed that only one particle can be carried by a micelle. That is, only one nucleation event is possible in each micelle.

The influence of the critical size on the formation of bimetallic nanoparticles in micelles was previously studied [36,45]. In this paper, n* was kept constant (*n*_A_* = *n*_B_* = *n*_A-B_* = 1) so as not to interfere in the discussion.

#### Nanoparticle growth

It is assumed that nanoparticles grow by deposition of metallic atoms on the nucleus, that is, a growing particle is built up layer by layer. Therefore, whenever a metal atom is exchanged towards a nucleated micelle, this atom is deposited on the nucleus. The sequence of metal deposition (Au or Pt) is stored in each micelle at each step.

In addition, nanoparticles can grow by autocatalysis [23,34,46]. This type of growth appears at advanced stages during the synthesis when collision between micelles containing reactants and a growing particle becomes frequent. An autocatalytic reduction is simulated by introducing two new criteria. When one of the colliding micelles is carrying a particle, the reduction will occur at double the rate and always at the existing particle. When both colliding micelles are nucleated, reduction takes place in the micelle containing the larger particle, and the atom is deposited onto it. These criteria allow us to include in the model the well-known belief that a bigger surface (bigger particle) has a greater probability to act as a catalyst. In this way, the growth of a preexistent nucleus is favored instead of the formation of a new one. To simplify, autocatalytic growth is governed by particle size, without taking into account the surface composition (Au or Pt).

Another type of growth is Ostwald ripening, that is, the growth of larger particles at the expense of smaller ones by transport of material. It is assumed that the easier solubilization of the smallest particles causes their decrease in size. These atoms/molecules, free in solution, will deposit on the largest particles. That is, large particles grow even larger, drawing material from the smaller ones, which shrink. This possibility is included in the exchange protocol of particles described as follows.

#### Intermicellar exchange protocol of growing particles

The transfer of a growing particle to another micelle also containing a particle is possible whenever the comunication channel of the colliding micelles is sufficiently large. As mentioned before, the size of the channel is represented by the *f* parameter, which is associated with surfactant film flexibility. A collision between micelles that both contain a growing particle can result in a unidirectional transfer of material. Thus, the smaller particle always moves from the initial micelle to the micelle containg the larger particle. In this way, two particles in two colliding micelles give rise to a single particle. This exchange is only possible when the smaller particle contains less than *f* atoms (i.e., it can traverse intermicellar channel). This rule is defined simply by the particle size, thus the composition (Au or Pt) is not taken into consideration. All types of material exchange (reactants, isolated atoms and growing particles) are allowed to be exchanged during the same collision.

#### Micelle size

The nanoparticle growth may be restricted by the size of the micelles because the surfactant film covering the micelle has a finite bending modulus. The simulation includes a micelle size parameter, which limits the nanoparticle size, establishing a maximum quantity of metal products which can be located inside the same micelle. In this study we present results using low values of reactant concentrations, thus the influence of micelle size on nanoparticle growth is assumed to be negligible.

#### Metal distribution in bimetallic nanoparticles

Nanoparticle formation is complete when the contents of each micelle do not vary with time. Each simulation run results in a set of micelles, each of which can contain a particle whose composition can be different. The composition of each particle is stored during the process. The final metal distribution in the nanoparticle is determined by the order in which the two metals are deposited onto the nanoparticle surface. Given the assumption that the particles are spherical, clustering steps give rise to the build up of concentric layers by adding new atoms over a particle. For each nanoparticle, the sequence of metals is stored and divided into ten concentric layers. The number of particles which contain a given percentage of Au is monitored from the inner layer (core) to the outer layer (shell), thus distribution and average composition (percent Au) can be calculated layer by layer. This final distribution is averaged over 1000 runs.

The nanoparticle structure is represented by histograms, in which the layer composition (% Au) is represented by a color grading. The distribution ranges from blue (0–10% for the fast metal Au) to red (90–100% of Au). 50% of each metal is represented by grey. Lighter colors represent a higher proportion of pure metal in the layer. The histograms show how many particles contain a given percentage of Au in each layer. In this way, the histograms allow analysis of the variation of metal arrangement from the early stages (core) as the synthesis reaches the shell formation. The nanoparticle structure is also represented by means of concentric spheres whose thickness is proportional to the number of layers for a given composition, keeping the same color scheme.

## Results and Discussion

### Metal distribution in Au–Pt nanoparticles for different Au:Pt ratios

**Figure 1:**
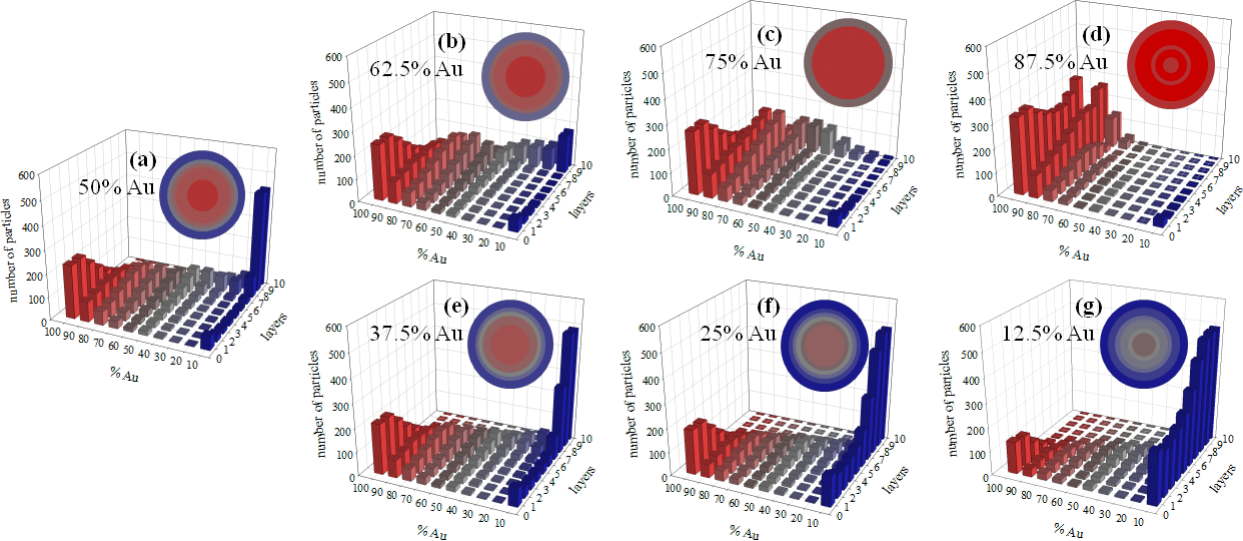
Histograms reprensent the number of particles with a given percentage of Au in each layer, from the nanoparticle core to the surface. Synthesis conditions: reduction rate ratio (v_Au_/v_Pt_ = 10), film flexibility (*k_ex_* = 5, *f* = 30) and average reactant concentration (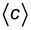 = 64 metal ions in a micelle). Each histogram shows a different proportion between metals salts: a) 50% Au, 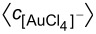 = 64, 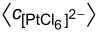 = 64; b) 62.5% Au, 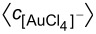 = 80, 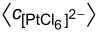 = 48; c) 75% Au, 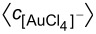 = 96, 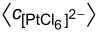 = 32; d) 87.5% Au, 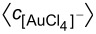 = 112, 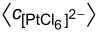 = 16; e) 37.5% Au, 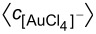 = 48, 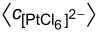 = 80; f) 25% Au, 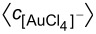 = 32, 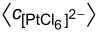 = 96; g) 12.5% Au, 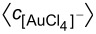 = 16, 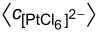 = 112 metal ions in a micelle. Scheme color: blue (0–45% of Au), grey (45–55% of Au), red (55–100% of Au). Brighter red corresponds to more Au. The circles in each histogram represent the nanoparticle structure in concentric layers, keeping the same color scheme.

A set of experiments were carried out varying the proportion between Au and Pt salts. Due to the complex mechanism governing the reactions inside the micelles, most of the parameters have been kept fixed in order not to interfere with the computational study. The following parameters are fixed: average metal concentration inside the droplets 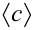 = 64 metal precursors per micelle, microemulsion composition (characterized by the exchange parameter, *k_ex_* = 1 and the film flexibility parameter *f* = 30), and critical nucleus sizes *n*_A_* = *n*_B_* = *n*_A-B_* = 1. The distribution of both metals within a nanoparticle can be observed in Figure 1, which shows the structures obtained by the simulation. When the quantities of the metal salts are equal (see Figure 1a, 50% Au), the nanoparticle shows a core–shell structure due to the difference in reduction rates (v_Au_ = 10∙v_Pt_). Most particles have a core composed by the faster reduction metal Au (see red bars on the left), followed by mixed middle layers, and then outer layers composed by Pt forming the shell (see blue bars behind on the right). As the Au proportion is larger (see the top row of Figure 1), the amount of Pt in the shell progressively diminishes until it disappears. Thus, 62.5% Au gives rise to a particle with a Pt-enriched shell (see Figure 1b), 75% Au gives rise to a mixed outer layer (see Figure 1c), and 87.5% Au particle does not result in a shell at all. Although Pt is mainly located in the outer layers, a few particles whose statistical weight is negligible result in a Pt core (see blue bars in front on the right).

With the decrease of Au proportion (see Figure 1e, f and g), the number of outer layers with pure Pt composition becomes higher, that is, the thickness of the Pt shell increases. This is an expected result, because if Pt reduces later, the resulting Pt atoms will deposit forming the more external layers. The higher the amount of Pt salt, the thicker the Pt shell. Regarding the cores, it is interesting to point out that the number of particles with a Pt core increases with the increase of % Pt (see blue bars in front on the right in bottom file). That is, when Pt is abundant (see Figure 1g, 12.5% Au) the number of particles with a pure Pt core is greater than the number of particles with a pure Au core. This is an unexpected result because it implies that an important amount of Pt is reduced at the very beginning of the synthesis, despite its slower reduction rate. It means that Au–Pt nanoparticles with a Pt-core can be obtained. Similar qualitative behavior is obtained when different average concentrations are simulated. This unpredicted evolution of metal segregation when the proportions are varied can be understood by means of a deeper kinetic study.

### Kinetic study

In micellar media, the diffusion of the reactants can be largely retarded or prevented [47]. Our hypothesis is that the resulting nanostructure is due to the particular combination of three main factors: the reduction rate ratio between both metals, the intermicellar exchange rate and the amount of each metal precursor inside micelles. These factors together will determine the particular sequence of deposition of the metals, which in turn will determine the metal distribution in the final nanoparticle.

**Figure 2:**
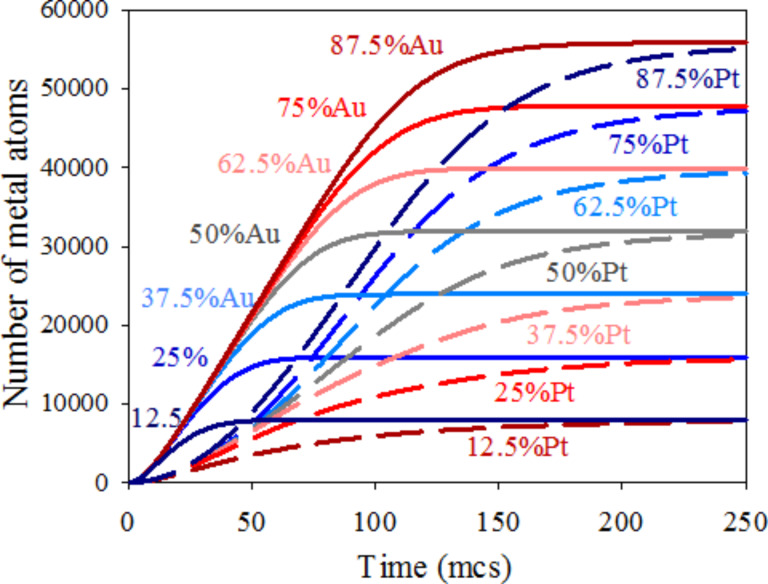
Time evolution of the number of metal atoms obtained in micelles. Continuous and discontinuous lines show the resulting Au and Pt, respectively. Synthesis conditions: flexible film (*k_ex_* = 5, *f* = 30), reduction rate ratio v_Au_/v_Pt_ = 10; average concentration 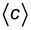 = 64. Scheme color: dark blue lines: 12.5% Au, 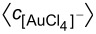 = 16, 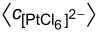 = 112; middle blue lines: 25% Au, 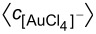 = 32, 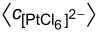 = 96; light blue lines: 37.5% Au, 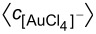 = 48, 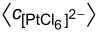 = 80; grey lines: 50% Au, 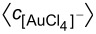 = 64, 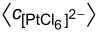 = 64; light red lines: 62.5% Au, 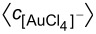 = 80, 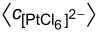 = 48; middle red lines: 75% Au, 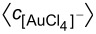 = 96, 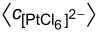 = 32; dark red lines: 87.5% Au, 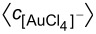 = 112, 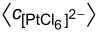 = 16.

As the synthesis advances, the simulation monitors the number and type of each atom as a function of time. Figure 2 shows the number of Au and Pt atoms produced in all micelles as the reaction proceeds. This figure corresponds to the same synthesis conditions shown in Figure 1. The continuous lines in Figure 2 represent the number of Au atoms produced at different initial Au proportions and discontinuous lines correspond to Pt atoms. Each Au curve (continuous line) has a corresponding Pt curve (discontinuous line in the same color): for example, an initial concentration 
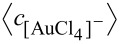
 = 96, 
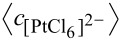
 = 32 is represented as a medium shade of red (75% Au, continuous line, and 25% Pt, discontinuous line). It can be clearly observed that the Au (which is reduced faster) is obtained earlier, even when the Au is the minority metal (compare the 12.5% Au and the 87.5% Pt lines). All curves lead to a plateau when the reactants have been exhausted. As initial metal salt amount is increased this plateau is reached at later stages of the synthesis, as expected. One interesting observation from these results is that the slopes of all the Au curves are similar. On the contrary, the Pt curves are strongly dependent on the initial Pt quantity. This implies that the influence of metal quantity on the actual metal production rate is different for Au than for Pt, as will be discussed later. Similar qualitative behavior was found using different average concentration values.

**Figure 3:**
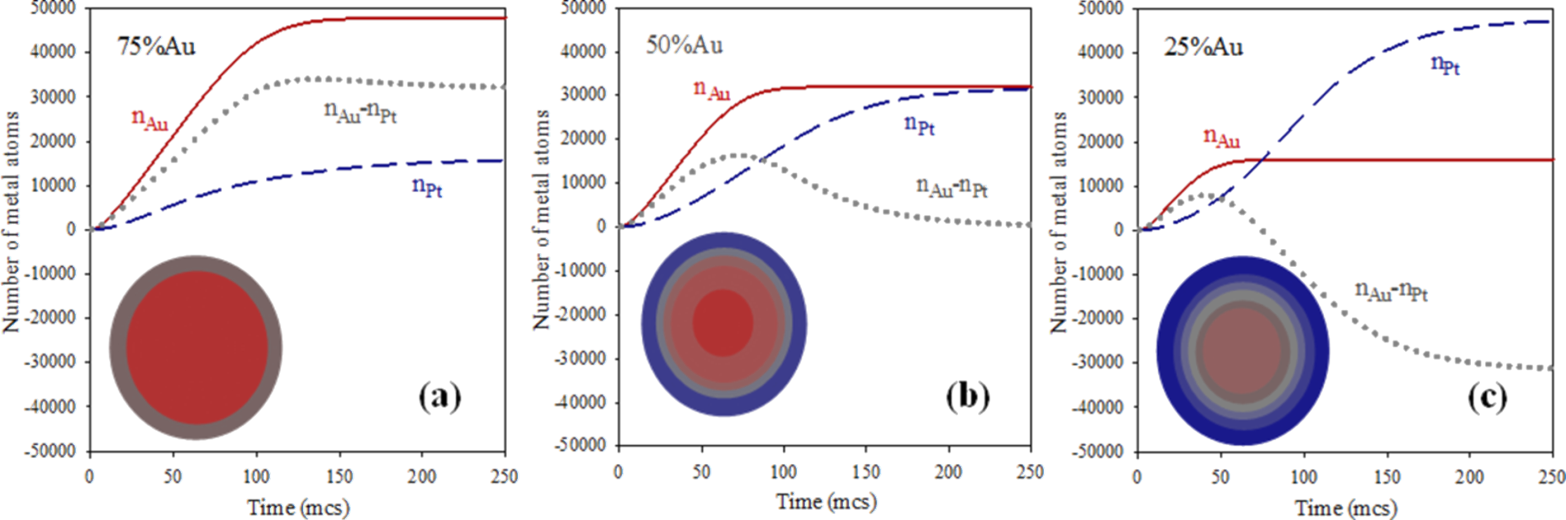
Time evolution of the number of metal atoms obtained in micelles. Continuous and discontinuous lines show the resulting Au and Pt, respectively. Dotted lines represent the difference between the number of Au and Pt atoms. Synthesis conditions: flexible film (*k_ex_* = 5, *f* = 30), reduction rate ratio v_Au_/v_Pt_ = 10, average concentration 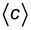 = 64. a) 75% Au, 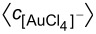 = 96, 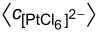 = 32; b) 50% Au, 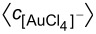 = 64, 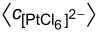 = 64; c) 25% Au, 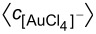 = 32, 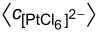 = 96.

The first aspect to discuss is how to relate the resulting metal curves to the nanoparticle structure. For a better understanding of the metal segregation in a nanoparticle, Figure 3 shows 25%, 50% and 75% Au curves with the corresponding Pt curves (the same as Figure 2). The dotted black line shows the difference between the number of Au and Pt atoms (n_Au_ − n_Pt_) as a function of time for each Au proportion. This difference increases as time goes on until a maximum is reached when the Au salt is almost exhausted. From this point, if Pt salt is also exhausted (see Figure 3a, 75% Au) due to the small amount of Pt, the n_Au_ − n_Pt_ curve remains aproximately constant. If the quantity of Pt salt is larger (see Figure 3b, 50% Au), the Pt continues to produce when the maximum is reached, and the difference diminishes until it becomes insignificant when slow Pt reduction finishes. Finally, if the minority metal is Au (see Figure 3c, 25% Au), thus Au finishes earlier, while Pt reduction continues. Consequently, the maximum is also reached earlier, and the difference n_Au_ − n_Pt_ takes negative values as times goes on. As the Au proportion diminishes, the maximum is not only displaced towards earlier stages but also the magnitude diminishes. This maximum reflects the largest difference in metal product availability, therefore its temporal localization and its magnitude will be directly correlated to the nanoparticle structure. Therefore, if a quite pronounced maximum appears at the beginning of the synthesis, a large quantity of Au atoms are obtained when the core is forming, and the core will be essentially homogeneous and composed of Au. From the maximum, Pt and the remaining Au are deposited on the core, giving rise to the middle mixed layers. In this case, a pure Pt shell is formed when the Au is exhausted. This is the case shown in Figure 3b, which corresponds to the core–shell structure obtained using 50% Au. A high maximum located at later stages as shown in Figure 3a (75% Au) implies the formation of many Au-composed layers. In this case, the fact that curve does not decay after the maximum is reflected in the mixed shell, that is, there is not enough Pt in the reaction media to build up an enriched Pt shell (see also Figure 1c). Finally, the short and early maximum shown in Figure 3c (25% Au) gives rise to a particle in which the small quantity of Au is mainly located in the inner layers, as showed in Figure 1f.

**Figure 4:**
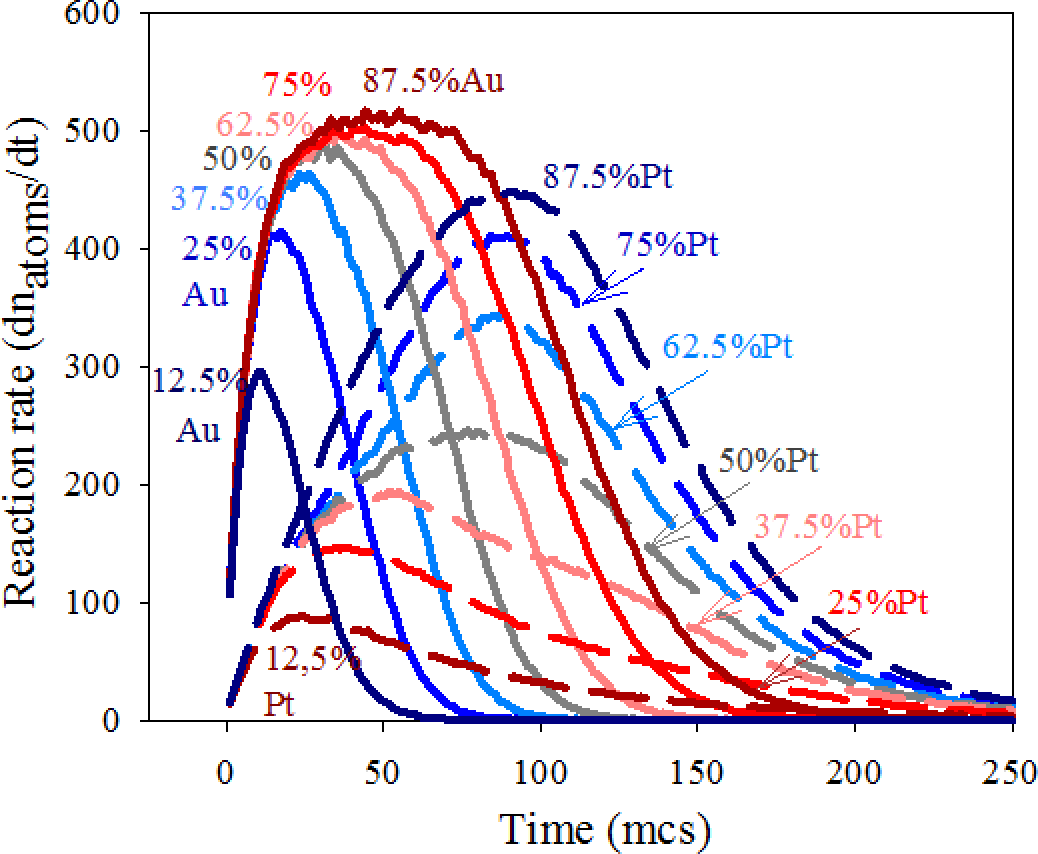
Reaction rate versus time. Continuous and discontinuous lines show the resulting Au and Pt, respectively. Synthesis conditions: flexible film (*k_ex_* = 5, *f* = 30), reduction rate ratio v_Au_/v_Pt_ = 10; average concentration 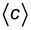 = 64. Scheme color: dark blue lines: 12.5% Au, 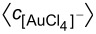
= 16, 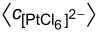 = 112; medium blue lines: 25% Au, 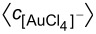 = 32, 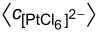 = 96; light blue lines: 37.5% Au, 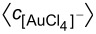 = 48, 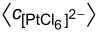 = 80; grey lines: 50% Au, 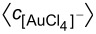 = 64, 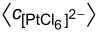 = 64; light red lines: 62.5% Au, 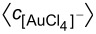 = 80, 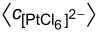 = 48; medium red lines: 75% Au, 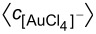 = 96, 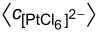 = 32; dark red lines: 87.5% Au, 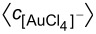 = 112, 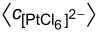 = 16 reactants per micelle.

A second aspect to study is understanding why the Pt reduction rate is more affected by an increase in reactant quantity than the Au rate. This implies that the influence of metal amount on the actual metal production rates is different in Au than in Pt. To gain more insight into how confinement influences chemical reactivity, the reaction rate of each metal was calculated from the slopes of the curves shown in Figure 2 as time progresses. In this way, both contributions (intermicellar exhange rate and chemical reduction rate) are taken into account to determine the reaction rate (dn_metal_/dt). To explain the results clearly, the chemical reduction rates (deduced from the standard potentials as v_Au_/v_Pt_ = 10) must be distinguished from the reaction rates (calculated from simulation data as dn_metal_/dt). Continuous and discontinuous lines in Figure 4 represent the resulting Au and Pt reaction rates, respectively, at different % Au, as the synthesis advances. The most impressive result in this figure is the profile of the rates, that is, an usual decay versus time is preceded by an increasing rate until a maximum is reached. To understand this result, one must keep in mind that the first requirement, before chemical reduction is possible, is the localization of the two reactants inside the same micelle.This implies an intermicellar exchange of material, whose rate is determined by microemulsion composition. The rate of the chemical reduction inside a micelle not only depends on the reduction rate but also on the rate of the intermicellar exchange. A priori, one could think that the intermicellar exchange rate equally affects both chemical reductions. But the interplay between exchange rate and reduction rate depends on the particular metal, as shown in Figure 4. In all cases it can be observed that the greater the metal amount, the quicker the actual reduction rate, as expected. However, the behavior of Au and Pt as increasing the corresponding metal amount is very different. Before the maximum, the Pt reaches a progressively faster rate as the Pt amount increases. On the contrary, the slope of Au rate does not depend on % Au and seems to reach a threshold from which it cannot increase anymore. In relation to the decay, Pt and Au also display different behavior. The decay slopes are determined by % Pt, but remain almost constant in the case of Au.

Firstly, we will discuss the Au reaction rate (see continuous lines in Figure 4). Previous results suggested that the compartmentalization of reaction media causes the Au reduction to be mainly controlled by the intermicellar exchange rate. The reason is that if reduction is very fast (as the case of Au), the slower step is the material intermicellar exchange, which allows for the reactants to interact. At this point, all Au precursors confined in the same micelle are reduced rapidly. In the simulation model, the intermicellar exchange rate is simulated by the parameter *k_ex_*, which quantifies how many ions/molecules can be transferred between colliding micelles. In this study, we present results obtained by allowing a maximum intermicellar exchange of five reactants (Au salt, Pt salt and/or reducing agent). That is, independent of the Au amount inside the micelle, only *k_ex_* = 5 reactants are allowed to be exchanged in each collision. As a result, although an instantaneous reaction is simulated for Au reduction, only a maximum of five Au atoms can be obtained during each effective collision. This restriction allows us to understand the Au rate profile. During the first steps (which determine the core formation), the reaction rate increases until it reaches the intermicellar control. Then, the Au reaction rate decreases as the Au salts are finished. Some time is needed to reach the intermicellar control. At the beginning of the synthesis, microemulsions containing the reactants are mixed. Due to Brownian motion, micelles collide with each other, but only collisions between one micelle containing Au salt (M-Au) and another micelle containing the reduction agent (M-R) allow both reactants to be found inside the same waterpool, where the reduction then takes place. The rest of the possible collisions (M-Au and M-Au, M-Au and M-Pt, M-Pt and M-Pt, M-Pt and M-R, M-R and M-R) can not give rise to Au atoms. These types of collisions simply redistribute reactants between micelles. As the reactants are redistributed between micelles, more collisions will be effective, providing metallic atoms and an increasing reaction rate. The speed at which the intermicellar control is achieved does not depend on the Au amount, but only on the material intermicellar exchange rate. As a consequence, all slopes at the beginning of the Au curves in Figure 4 are equal. If the Au amount is scarce, reaction rate does not reach intermicellar control due to the Au salt finishing earlier.

**Figure 5:**
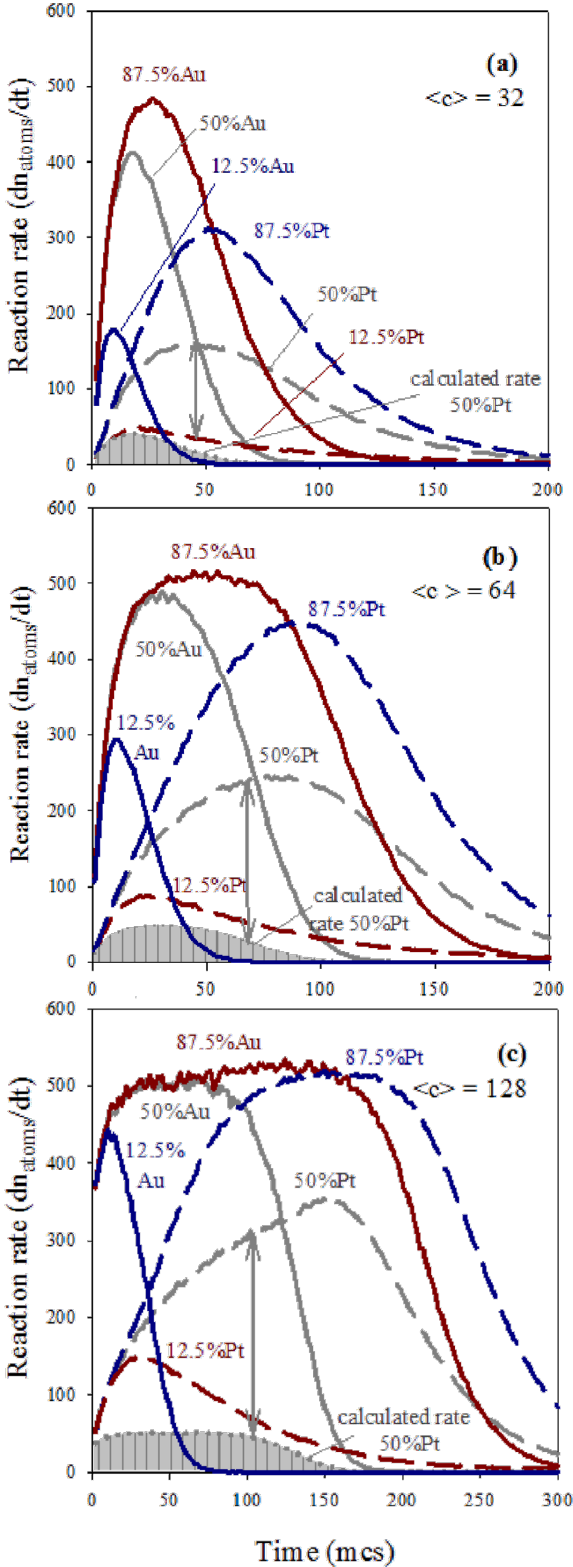
Reaction rate versus time. Continuous and discontinuous lines show the resulting Au and Pt, respectively. Synthesis conditions: flexible film (*k_ex_* = 5, *f* = 30), reduction rate ratio v_Au_/v_Pt_ = 10. Scheme color: dark blue lines: 12.5% Au, grey lines: 50% Au, dark red lines: 87.5% Au. Curves delimiting grey areas shows the calculated Pt rate as 0.10 times the Au reaction rate: v_Pt,calculated_ = 0.10∙dn_Au_/dt. a) average concentration 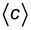 = 32 reactants per micelle (12.5% Au, 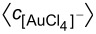 = 8, 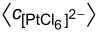 = 56; 50% Au, 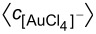 = 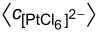 = 32; 87.5% Au, 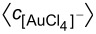 = 56, 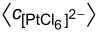 = 8); b) average concentration 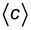 = 64 reactants per micelle (12.5% Au, 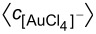 = 16, 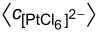 = 112; 50% Au, 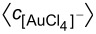 = 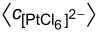 = 64; 87.5% Au, 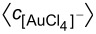 = 112, 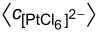 = 16); c) average concentration 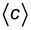 = 128 reactants per micelle (12.5% Au, 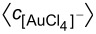 = 32, 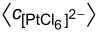 = 224; 50% Au, 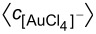 = 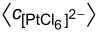 = 128; 87.5% Au, 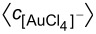 = 224, 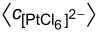 = 32).

Larger values of the reaction rate are reached by increasing the Au salt quantity, as expected. In addition, when the Au reaction rate achieves the exchange rate, it remains constant even though the Au amount inside the micelles would be larger (compare the curves at higher % Au). Likewise, according to our results, the decrease in rate is also not influenced by the Au amount, because once the Au salt is redistributed, the Au reduction only depends on the intermicellar exchange rate, that is, it steadily diminishes while there is Au precursor present. To summarize, due to the intermicellar exchange restrictions, the Au reaction rate is controlled by the material intermicellar exchange. Similar qualitative behavior was obtained using different total concentrations*,* as shown in Figure 5. The combination of a low average concentration and a low Au proportion leads to an initial Au quantity smaller than *k_ex_*, (see Figure 5a, 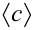 = 32, dark blue continuous line: 12.5% Au, 
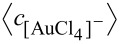
 = 4, 
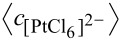
 = 28), and the initial slope does not reach the exchange control and the reactant concentration appears to be the controlling factor, as expected. Likewise, it can be observed that for a given proportion of reactants, faster reaction rates are reached at a higher concentration (compare Figure 5 a, b and c at a fixed % Au). In addition, as concentration increases, the plateau is reached at lower % Au and remains longer (see Figure 5c, 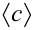 = 128, at higher % Au). Finally, it is interesting to emphasize that if synthesis conditions lead to the plateau achievement, the classical belief that the larger the reactant amount, the faster the reaction rate is not valid in this confined media.

Secondly, the reaction rate of the Pt reduction is strongly dependent on the Pt amount. Discontinuous lines in Figure 4 show that the higher the Pt proportion, the larger the reaction rate of Pt during all the synthesis. The rate of intermicellar exchange is the same for both metals, so the reason for this different behavior is not obvious. Previous studies suggest that micelles may act as a cage [27,48]. This assumption is based on the well-known cage effect in chemical kinetics. It is assumed that encounters (the process in which both reactants diffuse together to become neighbors) take place in a different way in a solution than in a gas. In a solution, the reactant molecules will generally jump from hole to hole in the solvent matrix. Occasionally, reactants will find themselves in the same solvent cage, so the two reactants can eventually form an encounter pair. The encounter pair can fail to react the first time, but it has many more opportunities as long as it remains in the same solvent cage. On the contrary, if the reaction takes place in the gas phase, the molecules have the freedom to go anywhere, thus many collisions will be encountered in pairs. However, if the collision should fail to be energetically or geometrically viable, the reactant molecules move away and are unlikely to meet again soon. Our hypothesis is that this approach can be used to compare the chemical kinetics in micelles and in a solution, instead of in liquid and in gas phases. That is, micelles allow for less encounters, but the reactants stay near each other for much longer than in a solution (in which there would be more encounters, but shorter time together). This assumption provides an explanation for the dependence of the Pt reaction rate on the reactant proportion. Although the intermicellar exchange rate is the same for both reactants, the Pt reduction rate is slower than the Au reduction rate. Only 10% of reactants (Pt salt and reducing agent) carried by the same micelle react to produce Pt atoms in each collision. The remaining 90% that did not react, stay in the micelle, giving rise to a reactant accumulation. The implications of this increase in concentration inside the micelles determine the kinetics of the Pt reduction. Although only a 10% of the reactants react, the amount of pairs of reactants inside the same micelle which are available to react is much higher than the pairs exchanged during the last collision. So the Pt reaction rate not only depends on the exchange constant (as the case of Au) but also on the reactant accumulation. As a result, the Pt reactant confinement will strongly affect the Pt reduction rate. This is because while there is enough reactant inside a micelle, the Pt reduction proceeds without depending on a new intermicellar exchange. This results in an increase of the Pt reduction rate due to the cage-like effect. This cage-like effect does not concern the Au reduction, because Au precursors are not accumulated due to an instantaneous reduction. On this basis, the increasing slopes before the peak of the discontinuous lines in Figure 4 can be explained by taking into account that more reactants (higher % Pt) lead to more accumulation of Pt, so the cage-like effect becomes more pronounced as % Pt increases. Therefore, if the Pt precursor amount is large enough, Pt curves must reach the intermicellar control. This behavior can be observed in the blue discontinuous line (87.5% Pt) in Figure 5c, which shows results using a larger average concentration (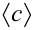 = 128 reactants per micelle).

After the peaks in Figure 4, the fact that the slopes of the Pt reactions rates strongly depend on % Pt (in contrast with Au) is also accounted for by the cage effect. At later stages of the synthesis, local accumulation of reactants inside the micelles diminishes because reactants have been finished as reaction proceeds. So the Pt reaction rate continuously decays, and the rate of this decay will be faster as the reactants are consumed faster. Then, if the cage-like effect takes place to a greater extent at higher % Pt, it also results in a quicker decay of Pt reaction rate.

Further evidence of the acceleration of Pt reduction in micelles can be achieved if the rate of Pt reduction is theoretically calculated for the case of the cage-like effect not taking place. By assuming that the Au reduction is as quick as the intermicellar exchange allows, the Au reaction rate (dn_Au_/dt) showed in Figure 4 will reflect the intermicellar dynamics, which does not depend on the kind of exchanged metal salt. If the Pt reduction is ten times slower than that of Au, and no Pt precursors accumulate inside micelles, then theoretically, the Pt reaction rate should be ten times slower than the Au reaction rate. A theoretical Pt reaction rate, calculated as v_Pt,calculated_ = 0.10∙dn_Au_/dt, would take into account the restrictions due to the intermicellar rate (reflected in dn_Au_/dt) without considering reactant accumulation. This theoretical Pt reaction rate is shown as the curve delimiting the grey filled areas in Figure 5. This estimated Pt rate can only be calculated by equal proportion of Au and Pt salts (50% Au), so that the initial quantity of metal salt does not interfere in discussion. Lines with double arrow are drawn to associate the theoretical Pt rate with the Pt reaction rate obtained from simulation data. Our assumption is that the large difference between both curves is due to the cage-like effect. The resulting gap is larger as concentration is higher, as is expected.

Summarizing, reactant confinement affects slow and fast reducting metals in different ways. The reaction rate of Au (faster metal) is controlled by the intermicellar exchange rate, so that once the exchange control is reached, the Au rate does not vary with the Au salt proportion. However, the reaction rate of Pt (slower metal) is strongly dependent on the Pt salt proportion during the entire synthesis due to the cage-like effect.

### Chemical kinetics and metal arrangement

The interplay between the reduction rates of both metals and the compartmentalization of the reaction media have key repercussions on the sequence of metal reduction. The resulting difference in reaction rates enables us to explain the modifications in metal segregation.

Due to the difference in the reduction rates, 50% Au gives rise to the typical core–shell structure. As % Au is increased, the quantity of Pt available to form the shell diminishes, and the Pt-enrichment in the shell progresively disappears.

When the % Pt is increased, the Pt reduction rate increases because of higher Pt quantity and the resulting cage-like effect. Consequently, more Pt is reduced sooner forming the inner layers, as clearly observed in Figure 1. More particles have a core composed of Pt as the % Pt is higher (see increasing blue bars in the inner layers in Figure 1, from 50 to 125% Au).

It is important to highlight that Au primarily comprises the core, even at the lowest Au percentages. This means that the acceleration of Pt reduction (due to a higher Pt quantity and the cage-like effect) cannot overtake the quicker Au reduction rate, even when a high % Pt is used.

A high % Pt gives rise to the formation of more external layers composed of Pt, so the thickness of the Pt shell increases with % Pt. This is a consequence of the excess of Pt, because once all of the Au salt is reduced forming the core, the nanoparticle can only grow by deposition of Pt.

## Conclusion

Intermicellar exchange is a factor of critical importance in metal reduction reactions in micelles, because its impact is different depending on the reduction rates of each metal. If the reduction rate is very fast, the intermicellar exchange will be the controlling step. Thus a change in the proportion of reactants inside the micelles would weakly affect the reaction rate. In contrast, the repercussions of intermicellar exchange rate on slower reduction metal strongly depend on the reactant proportion due to a cage-like effect. In this way, the reaction rate of the slower metal can be manipulated by changing reactant proportions. As a nanoparticle is build up by bringing together new layers according to the order of metals reduction, the relative reaction rates of both metals determine the metals arrangement. As a result, maintaining the average concentration and microemulsion composition, the metal distribution in a Au/Pt bimetallic nanoparticle can be manipulated just by varying the precursors proportion. For example, a core–shell structure is obtained using a proportion 1:1, a thick Pt shell with an enriched Pt core is obtained using higher Pt proportion, and pure Au covered by a mixed Pt-Au shell results if the precursors are mostly Au salts. These conclusions can also be applicable to other metals whose difference between standard reduction potentials is about 0.2–0.3 V. These results are very promising for the design of the experimental conditions to obtain a nanoparticle with a specific distribution of metals.
